# Previous caesarean delivery and the presence of caesarean scar defects could affect pregnancy outcomes after in vitro fertilization frozen-thawed embryo transfer: a retrospective cohort study

**DOI:** 10.1186/s12884-022-05085-0

**Published:** 2022-10-13

**Authors:** Yinfeng Zhang, Dominique de Ziegler, Xinyu Hu, Xiaomei Tai, Ying Han, Junfang Ma, Yunshan Zhang, Haining Luo

**Affiliations:** 1 Tianjin Central Hospital of Obstetrics and Gynecology/Nankai University Affiliated Maternity Hospital, Tianjin Key Laboratory of Human Development and Reproductive Regulation, No 156 Sanma Road, Nankai District, Tianjin, 300100 China; 2 Department of Obstetrics and Gynecology Hôpital Foch – Université de Paris Ouest (UVSQ), Suresnes France, France; 3 Department Obstetrics and Gynecology- NYU Langone Health, New York, NY USA; 4 Tianjin Medical University, Tianjin, 300070 China

**Keywords:** Caesarean delivery, Caesarean scar, In vitro fertilization, Frozen-thawed embryos, Live birth

## Abstract

**Background:**

Due to various iatrogenic and social factors, the global caesarean delivery (CD) rate has risen sharply in the past 30 years. It is more complicated and dangerous for women with a scarred uterus to experience pregnancy again than for women with a previous vaginal delivery (VD). In this study we investigated the impact of previous caesarean delivery (CD) and caesarean scar defects (CSDs) on pregnancy outcomes after in vitro fertilization frozen-thawed embryo transfer (IVF-FET).

**Methods:**

We conducted a retrospective cohort study that included 1122 women aged < 40 years who had a history of only one parturition (after 28 weeks of pregnancy) and who underwent their first FET cycle between January 2014 and January 2020. Patients were divided into the CD group, VD group, and CSD group. Thereafter, according to the number of transferred embryos, the CD, VD, and CSD groups were divided into the single embryo transfer (SET) group and the double embryo transfer (DET) group. Outcome measures in this study were live birth, clinical pregnancy, multiple pregnancy, ectopic pregnancy, pregnancy loss, pregnancy complications, preterm birth, and neonatal birth weight. Multivariate logistic regression was performed to evaluate the relationship between pregnancy outcomes and CD.

**Results:**

In SET patients, the clinical pregnancy and live birth rates were decreased in the CSD group compared with the VD and CD groups. In DET patients, the clinical pregnancy and live birth rates were significantly lower in theCSD group than in the CD and VD groups. After adjustment for confounders, previous CD and CSD were associated with a significantly lower clinical pregnancy rate and live birth rate than previous VD in the total sample. This effect was observed in DET patients, but not in SET patients. Additionally, DET patients with previous CD had a significantly higher multiple pregnancy rate (AOR = 0.47, 95% CI = 0.29, 0.75, *P* = 0.002) than those with previous VD, but no significant associations were observed in CSD and multiple pregnancies (AOR = 0.55, 95% CI = 0.23, 1.34, *P* = 0.192) between DET patients with CD and those with VD after adjusting for potential confounders.

**Conclusions:**

Our study showed that during an FET cycle, previous CD and the presence of a CSD could negatively affect pregnancy outcomes especially in DET patients.

## Introduction

Caesarean delivery (CD), which is a delivery method used after the occurrence of obstetric complications can reduce mortality rates among mothers and newborns. Proper use of CD plays an important role in reducing maternal and perinatal infant mortality and morbidity [1–3]. However, due to various iatrogenic and social factors, the global CD rate has risen sharply in the past 30 years [4–6]. It is more complicated and dangerous for women with a scarred uterus to experience pregnancy again than for women with previous vaginal delivery (VD) [7]. In addition, CD is associated with many complications, including caesarean scar defect (CSD), which is also known as an isthmocele, uterine transmural hernia, diverticulum, pouch, and niche [8].

CSD is characterized by defective myometrial healing at the site of the caesarean incision and commonly causes postmenstrual spotting, dysmenorrhea, chronic pelvic pain, dyspareunia, and infertility [9, 10]. Hysterosalpingography, transvaginal sonography (TVS), saline infusion sonohysterography, hysteroscopy, and magnetic resonance imaging can be used to diagnose CSD [11]. A meta-analysis reported that CD has a detrimental effect on the clinical pregnancy rate (CPR) and live birth rate (LBR) and increases the miscarriage rate (MR); CD is also associated with difficult embryo transfer after previous VD [12]. A previous study reported that CD without defects does not decrease the live birth rate after IVF compared with previous VD. However, the presence of CSD in women, especially young women (age ≤ 35 years), significantly impairs the chances of subsequent pregnancy in patients undergoing IVF-ET [13]. The effect of CD on IVF pregnancy outcomes is uncertain and only a few studies to date have evaluated the association between CD/CSD and frozen-thawed embryo transfer (FET) pregnancy outcomes. Studies have mainly focused on fresh in vitro fertilization/intracytoplasmic sperm injection (IVF/ICSI) cycles, in which the supraphysiologic hormonal milieu could lead to impairments in endometrial receptivity, uterine contraction, embryo implantation and placental development.

Therefore, this study used a retrospective cohort design to analyse the associations of previous CD and the presence of CSD with the pregnancy outcomes of patients undergoing FET. We also explored the effect of CD and CSD on reproductive outcomes in different patients undergoing single embryo transfer (SET) and double embryo transfer (DET).

## Methods

### Study design and population

**Fig. 1:**
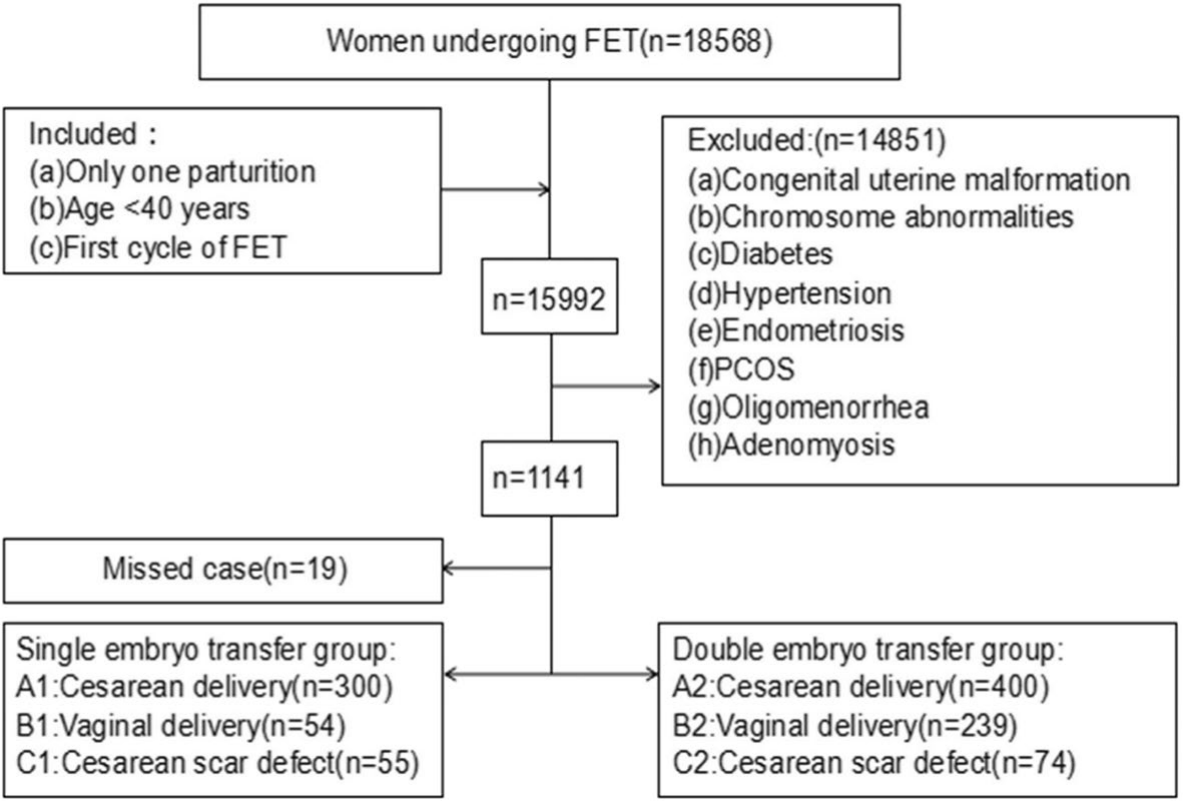
Flowchart of the study design

We conducted a retrospective cohort study of patients who received an FET cycle at the Tianjin Central Hospital of Obstetrics and Gynecology/Nankai University Affiliated Maternity Hospital from January 2014 to January 2020. The study design is shown in Fig. 1. The inclusion criteria were as follows: received their first FET cycle after a freeze-all policy, had a history of only one parturition (after 28 weeks of pregnancy) and were aged < 40 years. The exclusion criteria were as follows: congenital uterine malformation, chromosomal abnormalities in one or both spouses, diabetes or hypertension, oligomenorrhea or polycystic ovary syndrome (PCOS), endometriosis or adenomyosis, and missing clinical data.

All patients underwent TVS at least two times during the treatment. If TVS showed a pouch-like anechoic area with a depth ≥ 2 mm at the caesarean incision, the patient could be diagnosed with CSD [14, 15]. To ensure the reliability of the results, TVS for each patient was performed by at least two clinical professionals.

Patients who satisfied both the inclusion and exclusion criteria were divided into the following groups: the CD group, VD group, and CSD group. Thereafter, according to the number of transferred embryos, the CD, VD, and CSD groups were divided into the SET and DET groups.

### Endometrial preparation and embryo transfer

Based on each patient’s menstruation and clinical condition, a modified natural cycle or a hormone replacement therapy cycle was selected to prepare the endometrium. At our centre, 8-cell embryos with homogeneous or slightly uneven blastomere sizes, no fragments or fragments ≤10%, and divisions within the past 24 h were cryopreserved as D3 high-quality embryos; D5/D6 blastocysts of grade 3CC were cryopreserved. The embryos were frozen and thawed according to the instructions provided with the vitrified freezing/resuscitation solution (Japan Kato). Thawing and transfer of the embryo were performed 1 day after 3 or 5 full days of progesterone administration according to cleavage-stage or blastocyst-stage ET, respectively. Up to two embryos were transferred per cycle. Luteal support and oral dydrogesterone were prescribed for all patients after ET. TVS was performed 28 days after ET to confirm pregnancy.

### Variables

We collected the patients’ basic information, including age, body mass index (BMI), infertility duration, infertility factors, endometrial preparation, basal follicle-stimulating hormone (FSH), basal luteinizing hormone (LH), basal oestradiol (E2), number of retrieved oocytes, and the number of high-quality embryos transferred.

Basal FSH, LH and E2 are considered relevant covariates that affect pregnancy outcome. Women with elevated basal FSH who respond well and generate good-quality embryos have a chance of becoming pregnant and having a live birth [16, 17]. Combined FSH-LH was also shown to be directly correlated with embryo quality and implantation potential [18]. Mirkin et al. found that basal cycle day 3 serum E2 levels were independently and negatively associated with a significant decline in implantation rate. Others have proposed the basal cycle day 3 serum E2 level as an accurate IVF outcome predictor [19].

The primary outcome was live birth. We defined live birth as the delivery of a viable infant with signs of life after 24 completed weeks of gestation. The secondary outcomes were clinical pregnancy (gestational sac or a foetal heartbeat present using ultrasonography), multiple pregnancy, ectopic pregnancy, and pregnancy loss (amniotic sac present at 6–8 weeks after FET, no heartbeat before 28 gestational weeks [20], which was further categorized based on gestation length: early pregnancy loss (EPL) (≤13 weeks) and late pregnancy loss (LPL) (> 13 weeks)).Additional secondary outcomes were pregnancy complications, preterm birth (birth at 28–36^+6^weeks of gestation), and neonatal birth weight (low birth weight (LBW) (< 2500 g), high birth weight (HBW) (≥4000 g) and normal birth weight (NBW) (≥2500 g and < 4000 g)).

### Statistical analysis

The continuous variables are expressed as the mean and standard deviation (x̄±SD) and the categorical variables are expressed as percentages. Variables were assessed for normality to determine whether parametric or nonparametric statistical methods should be used. Differences between groups were tested by one-way analysis of variance (one-way ANOVA) with Fisher’s least significant difference (LSD) test as a post hoc test for continuous variables. The chi-square test was used for comparison of categorical variables with Bonferroni adjustment as the post hoc test. Multivariate-adjusted odds ratios (AORs) and corresponding 95% confidence intervals (CIs) were calculated using logistic regression models. Covariates included in multivariate models were selected by reference to previous studies, clinical significance and findings from univariate analyses (variables with a *P* value < 0.05 in the univariate model were selected). These covariates were age, prepregnancy BMI, infertility duration, infertility diagnosis, endometrial preparation, basal FSH, basal LH, basal E2, number of oocytes retrieved, and the number of high-quality embryos transferred. The model fitness in this study was tested by the Hosmer-Lemeshow goodness of fit test. Sensitivity analyses will be performed unadjusted and adjusted for the potential confounding covariates. *P* < 0.05 was considered to indicate statistically significant differences. The above statistical procedures were performed using SPSS version 22.0 (IBM Corp., Armonk, NY, USA).

We also calculated the statistical power using PASS version 11 (NCSS, LLC. Kaysville, UT, USA.). Considering α as 0.05 and the actual clinical pregnancy and live birth rates of each group (as shown in Table 2), the statistical power for detecting the differences in clinical pregnancy was 8% for CD vs. VD, 41% for CSD vs. VD among SET patients and was 31% for CD vs. VD and 58% for CSD vs. VD among DET patients. For live birth, the corresponding statistical power was 7, 18, 26 and 56% for the above mentioned groups.

## Results

In all, 1122 patients were retrospectively enrolled in this study. We followed up the patients for 1 year and obtained information on their clinical pregnancy outcomes. Among the 409 patients who underwent SET, 300 had previous CD, 54 had previous VD, and 55 had CSD. Their demographics and cycle characteristics are presented in Table 1. No significant differences were observed in these variables among the three SET groups. Among the remaining 713 patients who underwent DET, 400 had previous CD, 239 had previous VD, and 74 had CSD. Their demographics and cycle characteristics are presented in Table 1. The three DET groups exhibited significant differences in age, infertility duration and basal FSH.

**Table 1:** Demographics and cycle characteristics of patients in the SET and DET groups

	SET				DET			
	CD	VD	CSD	*P*-value^†^	CD	VD	CSD	*P*-value^†^
No. of patients	300	54	55		400	239	74	
Age (years)	33.5 ± 3.7	33.8 ± 3.9	34.9 ± 4.7^a^	0.051	33.7 ± 3.4	34.3 ± 3.5	35.5 ± 5.2^a,b^	0.000
Body Mass Index (kg/m2)	23.4 ± 3.2	23.2 ± 2.9	23.6 ± 3.0	0.813	23.1 ± 3.1	22.8 ± 2.9	22.5 ± 3.9	0.364
Duration of infertility (years)	4.2 ± 3.1	5.1 ± 3.2	4.4 ± 3.2	0.200	4.2 ± 3.1^b^	4.8 ± 3.3^a^	4.5 ± 3.2	0.035
Infertility diagnosis, n (%)				0.461				0.320
Male factor	47 (15.7)	9 (16.7)	4 (7.3)		70 (17.5)	38 (15.9)	10 (13.5)	
Tubal factor	118 (39.3)	18 (33.3)	17 (30.9)		137 (34.4)	101 (42.3)	28 (37.8)	
Other	75 (25.0)	12 (22.2)	17 (30.9)		105 (26.3)	55 (23.0)	21 (28.4)	
Unexplained	7 (2.3)	2 (3.7)	2 (3.6)		12 (3.0)	13 (5.4)	3 (4.1)	
Combined	53 (17.7)	13 (24.1)	15 (27.3)		76 (19.8)	32 (13.4)	12 (16.2)	
Endometrial preparation, n (%)				0.987				0.982
Modified natural cycle	178 (59.3)	32 (59.3)	32 (58.2)		244 (61.0)	144 (60.3)	45 (60.8)	
HRT	122 (40.7)	22 (40.7)	23 (41.8)		156 (39.0)	95 (39.7)	29 (39.2)	
Basal FSH (mIU/L)	6.4 ± 2.1	6.1 ± 2.4	6.7 ± 1.9	0.350	6.5 ± 1.8	6.3 ± 2.1	6.9 ± 2.2^b^	0.043
Basal LH (mIU/L)	4.3 ± 2.7	3.9 ± 2.7	4.0 ± 1.9	0.463	4.0 ± 2.2	3.9 ± 1.8	3.5 ± 1.4	0.212
Basal E2 (mIU/L)	44.7 ± 24.3	40.1 ± 16.3	48.4 ± 33.2	0.213	43.4 ± 26.2	46.4 ± 20.2	45.4 ± 20.2	0.294
No. oocytes retrieved	17.6 ± 8.5	17.2 ± 8.7	17.5 ± 10.5	0.858	16.9 ± 7.3	16.9 ± 7.4	15.0 ± 6.1	0.146
Number of high-quality embryos transferred, n (%)	253 (84.3)	41 (75.9)	47 (85.5)	0.282	295 (73.8)	190 (79.5)	55 (74.3)	0.249
Stage of embryo				0.811				0.583
Cleavage	246 (82.0)	44 (81.5)	47 (85.5)		377 (94.3)	221 (92.5)	68 (91.9)	
Blastocyst	54 (18.0)	10 (18.5)	8 (14.5)		23 (5.8)	18 (7.5)	6 (8.1)	

*HRT* Hormone replacement therapy, *E2* Estradiol, *FSH* Follicle-stimulating hormone, *LH* Luteinizing hormone, *SET* Single embryo transfer; *DET* Double embryo transfer, *CD* Previous cesarean delivery, *VD* Previous vaginal delivery, *CSD* Cesarean scar defect

^†^One way analysis of variance for continuous variables with LSD as post hoc test; chi-square test was used for categorial variables with Bonferroni adjustment in the post hoc test

^a^*P* < 0.05 compared with CD group; ^b^*P* < 0.05 compared with VD

The results of the unadjusted analyses are shown in Table 2. In SET patients, the clinical pregnancy and live birth rates were decreased in the CSD group compared with the VD and CD groups, but no significant differences were observed in any of the pregnancy outcomes among the three SET groups, except for neonatal birth weight (*P* = 0.0331). In DET patients, the clinical pregnancy and live birth rates were significantly lower in the CSD group than in the CD and VD groups (*P* < 0.001). Moreover, in DET patients, the multiple pregnancy rate was significantly lower in the CD group and CSD group than in the VD group. In DET patients, ectopic pregnancies only occurred in the CD group (five cases). No significant differences were found in the pregnancy loss rate, pregnancy complication rate, preterm birth rate, or neonatal birth weight among the three DET groups.

**Table 2:** Reproductive outcomes in the SET and DET groups

	SET				DET			
	CD	VD	CSD	*P*-value^†^	CD	VD	CSD	*P*-value^†^
No. of patients	300	54	55		400	239	74	
Clinical pregnancy, n (%)	117 (39.0)	24 (44.4)	15 (27.3)	0.1531	187 (46.8)^b^	143 (59.8)^a^	25 (33.8)^b^	< 0.001
Multiple pregnancy, n (%)	0	0	0	NA	43 (10.8)^b^	48 (20.1)^a^	7 (9.5)	0.0021
Pregnancy loss, n (%)	22 (18.8)	3 (12.5)	2 (13.3)	0.6921	33 (17.6)	24 (16.8)	7 (28.0)	0.3961
EPL, n (%)	20 (17.1)	3 (12.5)	2 (13.3)	0.8181	27 (14.4)	18 (12.6)	6 (24.0)	0.3241
LPL, n (%)	2 (1.7)	0	0	0.7131	6 (3.2)	6 (4.2)	1 (4.0)	0.8901
Ectopic pregnancy, n (%)	3 (2.6)	1 (4.2)	0	0.7261	5 (2.7)	0	0	0.1021
Pregnancy complications, n (%)	18 (15.4)	5 (20.8)	0	0.1881	36 (19.3)	21 (14.7)	5 (20.0)	0.5241
Preterm birth, n (%)	12 (13.2)	4 (21.1)	2 (15.4)	0.6751	33 (22.4)	20 (16.9)	6 (33.3)	0.2180
Live birth, n (%)	91 (30.3)	19 (35.2)	13 (23.6)	0.4141	147 (36.8)^b^	118 (49.4)^a^	18 (24.3)^b^	< 0.001
LBW, n (%)	6 (6.6)	0	0	0.3301	23 (13.5)	26 (17.3)	4 (20.0)	0.5511
NBW, n (%)	74 (81.3)	12 (63.2)	13 (100)^b^	0.0331	137 (80.6)	114 (76.0)	15 (75.0)	0.5721
HBW, n (%)	11 (12.1)^b^	7 (36.8)^a^	0^b^	0.0061	10 (5.9)	10 (6.7)	1 (5.0)	0.9351

*EPL* Early pregnancy loss (≤13 weeks), *LPL* Late pregnancy loss (> 13 weeks), *LBW* Low birth weight (<2500 g), *HBW* High birth weight (≥4000 g), *NBW* Normal birth weight (≥2500 g and <4000 g), *SET* Single embryo transfer, *DET* Double embryo transfer, *CD* Previous cesarean delivery, *VD* Previous vaginal delivery, *CSD* Cesarean scar defect

^†^Chi-square test was used for categorial outcomes with Bonferroni adjustment in the post hoc test

^a^*P* < 0.05 compared with CD group; ^b^*P* < 0.05 compared with VD

Logistic regression was performed to determine the effects of previous CD and the presence of CSD on clinical pregnancy, multiple pregnancy, pregnancy loss, preterm birth, and live birth while adjusting for age, prepregnancy BMI, infertility duration, infertility diagnosis, endometrial preparation, basal FSH, basal LH, basal E2，the number of high-quality embryos transferred and the number of oocytes retrieved as potential confounders. The results are presented in Table 3. Previous CD and CSD were associated with a significantly lower clinical pregnancy rate and live birth rate than previous VD in the total sample. However, this effect was observed in DET patients, but not in SET patients before or after adjusting for potential confounders. Additionally, DET patients with previous CD had a significantly higher multiple pregnancy rate (AOR = 0.47, 95% CI = 0.29, 0.75, *P* = 0.002) than those with previous VD, but no significant associations were observed in CSD and multiple pregnancies (AOR = 0.55, 95% CI = 0.23, 1.34, *P* = 0.192) between DET patients with CD and those with VD after adjusting for potential confounders.

**Table 3:** Multivariate logistic regression analysis of the patients who underwent subsequent frozen embryo transfer

	CD vs. VD				CSD vs. VD			
	AOR (95%CI)	***P***	AOR (95%CI) ^a^	***P***	AOR (95%CI)	***P***	AOR (95%CI) ^a^	***P***
**Total samples**								
Clinical pregnancy	0.58 (0.44,0.76)	**< 0.001**	0.57 (0.42,0.76)	**< 0.001**	0.34 (0.22,0.53)	**< 0.001**	0.39 (0.25,0.62)	**< 0.001**
Pregnancy loss	0.84 (0.52,1.36)	0.479	0.98 (0.6,1.62)	0.947	0.74 (0.34,1.62)	0.45	0.89 (0.4,1.99)	0.776
EPL	0.93 (0.55,1.59)	0.797	1.07 (0.61,1.86)	0.816	0.86 (0.37,1.99)	0.718	0.97 (0.41,2.31)	0.944
Live birth	0.59 (0.44,0.77)	**< 0.001**	0.56 (0.42,0.75)	**< 0.001**	0.36 (0.23,0.57)	**< 0.001**	0.41 (0.25,0.66)	**< 0.001**
Preterm birth	0.7 (0.42,1.17)	0.176	0.76 (0.44,1.3)	0.314	0.52 (0.21,1.31)	0.165	0.69 (0.27,1.79)	0.451
**SET samples**								
Clinical pregnancy	0.8 (0.45,1.43)	0.452	0.73 (0.39,1.36)	0.324	0.47 (0.21,1.04)	0.063	0.45 (0.19,1.06)	0.069
Pregnancy loss	1.35 (0.39,4.66)	0.64	1.14 (0.31,4.14)	0.846	0.64 (0.1,4)	0.634	0.54 (0.08,3.8)	0.537
EPL	1.21 (0.35,4.24)	0.761	0.99 (0.27,3.64)	0.984	0.64 (0.1,4)	0.634	0.54 (0.08,3.8)	0.532
Live birth	0.8 (0.44,1.48)	0.479	0.76 (0.4,1.46)	0.416	0.57 (0.25,1.32)	0.188	0.59 (0.24,1.43)	0.243
Preterm birth	0.52 (0.16,1.68)	0.275	0.5 (0.15,1.71)	0.271	0.47 (0.08,2.69)	0.398	0.58 (0.09,3.63)	0.557
**DET samples**								
Clinical pregnancy	0.59 (0.43,0.82)	**0.001**	0.56 (0.39,0.79)	**< 0.001**	0.34 (0.2,0.59)	**< 0.001**	0.4 (0.23,0.71)	**0.002**
Multiple pregnancy	0.48 (0.31,0.75)	**0.001**	0.47 (0.29,0.75)	**0.002**	0.42 (0.18,0.96)	**0.041**	0.55 (0.23,1.34)	0.192
Pregnancy loss	0.81 (0.46,1.4)	0.443	0.84 (0.47,1.48)	0.541	0.94 (0.39,2.27)	0.884	0.93 (0.37,2.32)	0.876
EPL	0.89 (0.48,1.65)	0.709	0.93 (0.49,1.76)	0.822	1.08 (0.41,2.84)	0.871	0.98 (0.36,2.65)	0.962
LPL	0.59 (0.19,1.85)	0.368	0.6 (0.18,1.98)	0.401	0.53 (0.06,4.49)	0.562	0.68 (0.08,6.14)	0.734
Pregnancy complications	1.03 (0.58,1.8)	0.927	1 (0.55,1.8)	0.995	0.75 (0.27,2.07)	0.581	0.98 (0.34,2.79)	0.967
Live birth	0.6 (0.43,0.82)	**0.002**	0.56 (0.4,0.79)	**0.001**	0.33 (0.18,0.59)	**< 0.001**	0.39 (0.21,0.72)	**0.003**
Preterm birth	0.87 (0.49,1.56)	0.643	0.91 (0.5,1.66)	0.756	0.59 (0.2,1.79)	0.353	0.81 (0.26,2.53)	0.714

*SET* Single embryo transfer, *DET* Double embryo transfer, *CD* Previous caesarean delivery, *VD* Previous vaginal delivery, *CSD* Caesarean scar defect, *EPL* Early pregnancy loss (≤13 weeks), *LPL* Late pregnancy loss (> 13 weeks), *OR* Odds ratio, *CI* Confidence interval

^a^Adjusted for age, prepregnancy BMI, infertility duration, infertility diagnosis, endometrial preparation, basal FSH, basal LH, basal E2, number of oocytes retrieved, and the number of high-quality embryos transferred

## Discussion

Our study showed that during an FET cycle, previous CD and the presence of CSD could negatively affect pregnancy outcomes after SET and DET. In the unadjusted analyses, the clinical pregnancy and live birth rates were decreased in the CSD group compared with the VD and CD groups in SET patients. In DET patients the clinical pregnancy and live birth rates were significantly lower in the CSD group than in theCD and VD groups. After adjustment for confounders, in DET patients, previous CD was associated with a significantly lower clinical pregnancy rate, multiple pregnancy rate and live birth rate than previous VD. Additionally, CSD was associated with a significantly lower clinical pregnancy rate and live birth rate than previous VD. CD and CSD could affect the pregnancy outcomes of patients after in vitro fertilization frozen-thawed embryo transfer, although the results were not significantly different in the SET group. This may be related to the small number of patients in the SET group.

Several previous studies have reported similar results. Naji et al. reported that the presence of a uterine scar affects the location of embryo implantation, and the mean distance between the embryo implantation site and the internal cervical ostium is 26.6 or 35.3 mm in women with previous CD or VD, respectively [21]. It has been reported in the literature that a caesarean section scar can reduce the chance of embryo implantation and lead to spontaneous abortion [22]. Incomplete uterine healing after a CD, termed a ‘niche’ can affect embryo implantation due to the presence of an embryotoxic environment, a mechanism similar to that proposed for hydrosalpinx [23, 24]. In addition, chronic inflammation caused by poor endometrial healing and menstrual blood stasis in diverticula affects endometrial receptivity and results, in difficult embryo implantation and an increased miscarriage rate after implantation [22]. Currently, no guidelines have been established for the treatment of CSD. The main methods to treat CSD are medical treatments (oral contraceptives and intrauterine devices with levonorgestrel) and surgical treatments (hysteroscopic resection, laparoscopic repair, and vaginal repair) [11]. These treatments can alleviate clinical symptoms and improve quality of life [25–27].

The advantages of this study included the strict inclusion criteria and the inclusion of patients with a history of only one parturition. Additionally, to accurately assess the effects of previous CD and the presence of CSD on pregnancy outcomes after IVF-FET, we excluded patients with various confounding factors that greatly affect pregnancy outcomes, such as PCOS, adenomyosis, and other diseases. This study used patients who underwent the first thawing cycle as the study population, which mitigates detrimental influences and provides a novel model to assess the sole impact of CD and CSD on embryo transfer. Analyses were restricted to first transfers in a freeze-all setting, thus minimizing the potential bias from embryo selection and repeated implantation failure. We used multivariate logistic regression to adjust for baseline characteristics that may differ among the three groups to reduce the influence of selection bias on the results.

The study also has certain limitations. First, it is a single-centre retrospective study. The overall sample size of the study, especially that of the SET group, was still limited, which reduced the statistical power. Statistical power was determined using PASS (Hintze, J. (2011). PASS 11. NCSS, LLC. Kaysville, UT, USA. www.ncss.com.). We had a statistical power of 50–60% to detect the differences in clinical pregnancy and live birth among patients in the DET group, while the statistical power was only approximately 10% in the SET group. Thus, the null association between CD/CSD versus VD might be due to the limited sample size. Although we reduced selection bias as much as possible, we still could not adjust for some known and unknown confounding factors. For example, we did not collect information on previous CD surgical methods, or residual myometrial thickness.

In summary, among patients who underwent IVF-FET, previous CD and the presence of CSD could reduce the rate of clinical pregnancy and live birth, especially in DET patients. With the implementation of the universal two-child policy in China, the fertility rate of women with uterine scars will increase. Findings from this study add further evidence that previous CSD negatively affects pregnancy outcomes. It is recommended to avoid medically unnecessary primary CD. For infertile patients with a history of CD, if they desire to have a second child through IVF, it is important to receive counselling before the first cycle begins. DET does not significantly improve the pregnancy outcome of patients, and thus SET is recommended for such patients.

## Conclusion

Our study showed that during an FET cycle, previous CD and the presence of CSD could negatively affect pregnancy outcomes especially in DET patients. Avoiding medically unnecessary primary CD and limiting the number of transfer embryos are recommended.

## Data Availability

The data used or analyzed during the current study are included within the article. The datasets are not publicly available due to the hospital policy and personal privacy. However, the datasets are available from the corresponding author on reasonable request.

## Abbreviations

CD: Cesarean delivery
IVF: In vitro fertilization
FET: Frozen-thawed embryo transfer
SET: Single embryo transfer
CSD: Caesarean section defect
VD: Vaginal delivery
DET: Double embryo transfer
TVS: Transvaginal sonography
EPL: Early pregnancy loss
LPL: Late pregnancy loss
PCOS: Polycystic ovary syndrome
BMI: Body mass index
LH: Luteinizing hormone
E2: Estradiol
ORs: Odds ratios
CIs: Confidence intervals
FSH: Follicle-stimulating hormone
